# Uses of Water in Modern Medicine

**Published:** 1892-10

**Authors:** 


A PRACTICAL MANUAL OF DISEASES OF THE SKIN, By Geo. H. Rohe, M. D., Baltimore, assisted by J. Williams Lord, A. B., M. D., pp. 303. Price $1 net. For sale by the F. A. Davis Co., publishers, Philadelphia, 1892.

A careful examination of this little book has convinced the writer that it is one of the best arranged and most practical of the small volumes on skin diseases now out. B.

